# Family Jars

**Published:** 1854-12

**Authors:** 


FAMILY JARS.

Jars of jelly, jars of jam,

Jars of pottled beef and ham,

Jars of early gooseberries nice,

Jars of mince meat, jars of spice,

Jars of orange marmalade,

Jars of pickles, all home-made,

Jars of cordial elder-wine,

Jars of honey, superfine;

Would that only jars like these,

Could be found in families!

